# Evidence for heterogeneity in China’s progress against pulmonary tuberculosis: uneven reductions in a major center of ongoing transmission, 2005–2017

**DOI:** 10.1186/s12879-019-4262-2

**Published:** 2019-07-12

**Authors:** Ting Li, Qu Cheng, Charles Li, Everleigh Stokes, Philip Collender, Alison Ohringer, Xintong Li, Jing Li, Jonathan L. Zelner, Song Liang, Changhong Yang, Justin V. Remais, Jin’ge He

**Affiliations:** 1 Institute of Tuberculosis Control and Prevention, Sichuan Center for Disease Control and Prevention, Chengdu, 610041 China; 2 Division of Environmental Health Sciences School of Public Health, University of California, Berkeley, 94720 USA; 3 Department of Biostatistics Rollins School of Public Health, Emory University, Atlanta, 30322 USA; 4 Department of Epidemiology and Center for Social Epidemiology and Population Health School of Public Health, University of Michigan, Ann Arbor, 48109 USA; 5 Department of Environmental and Global Health College of Public Health and Health Professions, University of Florida, Gainesville, 32611 USA; 6 Institute of Public Health Information, Sichuan Center for Disease Control and Prevention, Chengdu, 610041 China

**Keywords:** Tuberculosis, Spatial distribution, Cluster analysis, Descriptive epidemiology

## Abstract

**Background:**

China contributed 8.9% of all incident cases of tuberculosis globally in 2017, and understanding the spatiotemporal distribution of pulmonary tuberculosis (PTB) in major transmission foci in the country is critical to ongoing efforts to improve population health.

**Methods:**

We estimated annual PTB notification rates and their spatiotemporal distributions in Sichuan province, a major center of ongoing transmission, from 2005 to 2017. Time series decomposition was used to obtain trend components from the monthly incidence rate time series. Spatiotemporal cluster analyses were conducted to detect spatiotemporal clusters of PTB at the county level.

**Results:**

From 2005 to 2017, 976,873 cases of active PTB and 388,739 cases of smear-positive PTB were reported in Sichuan Province, China. During this period, the overall reported incidence rate of active PTB decreased steadily at a rate of decrease (3.77 cases per 100,000 per year, 95% confidence interval (CI): 3.28–4.31) that was slightly faster than the national average rate of decrease (3.14 cases per 100,000 per year, 95% CI: 2.61–3.67). Although reported PTB incidence decreased significantly in most regions of the province, incidence was observed to be increasing in some counties with high HIV incidence and ethnic minority populations. Active and smear-positive PTB case reports exhibited seasonality, peaking in March and April, with apparent links to social dynamics and climatological factors.

**Conclusions:**

While PTB incidence rates decreased strikingly in the study area over the past decade, improvements have not been equally distributed. Additional surveillance and control efforts should be guided by the seasonal-trend and spatiotemporal cluster analyses presented here, focusing on areas with increasing incidence rates, and updated to reflect the latest information from real-time reporting.

**Electronic supplementary material:**

The online version of this article (10.1186/s12879-019-4262-2) contains supplementary material, which is available to authorized users.

## Background

Tuberculosis (TB) is caused by inhalation of the bacterium *Mycobacterium tuberculosis* [1], and is the leading cause of death from a single infectious agent, ranking above HIV/AIDS, and as the tenth most common cause of death worldwide [2]. Pulmonary tuberculosis (PTB) contributed 85% of all notified TB cases worldwide, and 88% of TB deaths [2, 3]. Globally, China had the second largest number of new TB cases in 2017, accounting for 8.9% of all new cases worldwide [2]. The spatial distribution of PTB in the country is heterogeneous: incidence rates are higher in poorer, inland and western regions, and lower in more developed coastal regions [4–6]. Among the major foci of ongoing transmission in China, the southwestern province Sichuan has, until recently, experienced a very high TB burden, with estimated prevalence of active and smear-positive PTB of 598 and 104 cases per 100,000 persons in 2010, respectively—substantially higher than the corresponding national averages of 442 and 59 cases per 100,000 persons [7, 8]. With a population of more than 80 million people, the province accounts for 6.9 and 5.5% of China’s total PTB incidence and mortality [9].

Despite the high burden of PTB and a population of more than 80 million people, there is a limited understanding of the demographic and spatiotemporal distributions of PTB in the region, and thus information is limited on whether there are areas within the province that have seen limited reductions, or even increases, in transmission [10–12]. Here, we estimate annual PTB notification rates and the spatiotemporal distribution of PTB incidence in the region based on data from the National Infectious Disease Reporting System (NIDRS)—China’s real-time, high-coverage electronic disease notification system. We detail the demographic, temporal, and spatial distributions of reported active and smear-positive PTB cases from 2005 to 2017, and apply spatiotemporal scan statistics to detect areas with unusually high incidence rates in order to identify key areas where future control and prevention measures might be targeted.

## Methods

### Epidemiologic and covariate data

The study region of Sichuan Province is located in southwest China (26.05–34.32°N, 97.35–108.52°E), and ranks as the fourth most populous and fifth largest province in China [13, 14]. The region’s unique landscape, with low lying plains in the east and high-elevation mountains in the west, yields a dramatic east-to-west gradient in temperature (high-to-low), solar radiation (low-to-high), population density (high-to-low), and socioeconomic development and status (high-to-low), all of which have been examined with respect to their association with PTB [15–18].

Reported active PTB cases diagnosed between Jan 1, 2005 and Dec 31, 2017 were obtained from NIDRS—a passive electronic surveillance system whose coverage extends across almost all healthcare facilities in China [19]—and aggregated for each county and date. Upon diagnosis, all laboratory-confirmed, clinically-diagnosed, and suspect PTB cases are required to be reported to NIDRS within 24 h. Reporting of other types of TB, such as pleural TB and extra-pulmonary TB, is not mandated in China, and thus only PTB is considered in this study. NIDRS data include patient age, gender, occupation, residential address, date of onset, and date of diagnosis, as well as the name and location of the reporting facility. Because PTB is a chronic disease and patients’ recollection of the date of onset is often inaccurate (in our data, reporting is disproportionately high for the 1st, 10th, and 20th days of each month, see Additional file 1: Figure S1), we analyzed cases referenced to their date of diagnosis. Total number of reported HIV/AIDS cases from 2005 to 2017 was also obtained from NIDRS.

County-level year-end population data, stratified by sex and age, were obtained for 2005–2016 for the study region [20], and were projected for 2017 via exponential smoothing based on prior years’ population. Proportion of ethnic minorities was obtained from the 2010 population census [13, 21], and was assumed to be stable over the period of analysis. The predominant landform type (e.g., mountains, hills, plains) of each county was obtained from government data [22], and counties were roughly categorized as urban or rural based on the suffix of the county’s name in 2010: Qu (*区*) for urban counties or Xian (*县*) for rural counties. Annual mean maximum temperature was acquired for prefecture capitals from TerraClimate [23]; prefectures, which are the administrative level above counties in China, were then ordered by climate to examine seasonal patterns of PTB. Publically available administrative boundaries used for data visualization were obtained from China’s National Earth System Science Data Sharing Infrastructure [24].

### Case definition

**Fig. 1:**
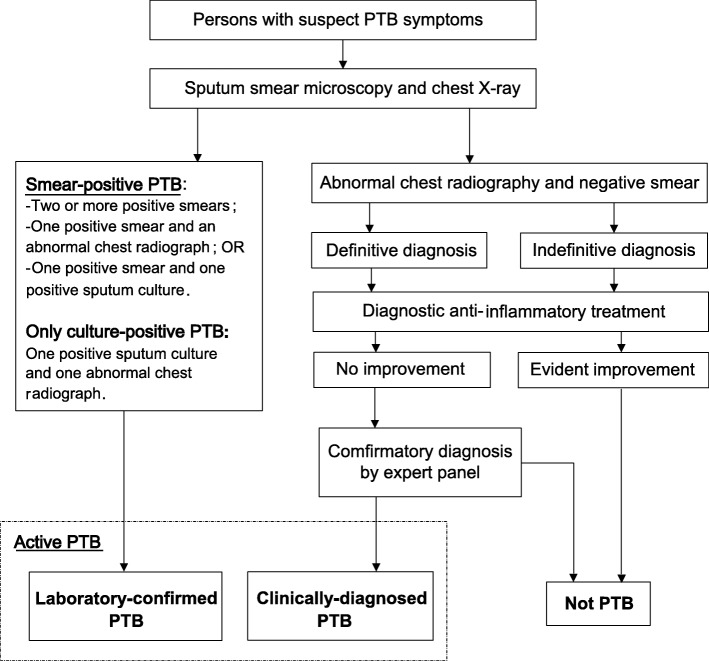
Flowchart for PTB diagnosis per China’s National Diagnostic Criteria for Pulmonary Tuberculosis [25]. PTB, pulmonary tuberculosis

Smear-positive and active PTB cases are diagnosed according to the National Diagnostic Criteria for Pulmonary Tuberculosis (WS288–2008) [25] (Fig. 1). Smear-positive cases are defined as patients with suspect PTB symptoms (e.g., cough for more than 2 weeks or haemoptysis) and at least one sputum smear with detectable acid-fast bacilli. Active PTB cases may be defined through laboratory-confirmation or clinical diagnosis. Laboratory-confirmation may be obtained via detection of mycobacteria during sputum smear microscopy, or their growth in culture. Clinical diagnosis requires an abnormal chest radiography result and the lack of responsiveness to diagnostic anti-inflammatory treatment (which excludes anti-TB drugs) [25].

### Data analysis

#### Seasonality and trend analysis

To explore seasonal trends in PTB incidence, we examined monthly and daily seasonality indices at the prefecture level, calculated as the ratio of observed case counts on a certain month or calendar day compared to the mean monthly or daily case count for the year. Because prior research indicated a negative correlation between temperature and the amplitude of province-level seasonal fluctuations in active tuberculosis cases in China [26], we ordered prefectures by temperature when examining seasonal trends.

To calculate the annual rate of change of reported PTB incidence rates at the county level, we fitted linear regressions to the trend component extracted from the monthly incidence rate time series by the seasonal-trend decomposition procedure based on LOESS (STL) [27]. We also applied linear regression to estimate the annual growth rate of reporting facilities per 100,000 persons. R statistical software [28] was used for time series analyses, regression analyses and for rendering heat maps.

#### Spatiotemporal cluster analyses

Spatiotemporal clusters were detected using both elliptic space-time scan statistics [29] and flexibly shaped spatial scan statistics [30]. The former is ill-equipped for detecting irregularly shaped clusters, while the current version of flexibly shaped scan statistics can only detect spatial, and not spatiotemporal, clusters (see Additional file 1: Supplementary methods). We used SaTScan [31] to implement elliptic space-time scan statistics, with a discrete Poisson probability model and maximum spatial and temporal cluster sizes set to be 50% of the population at risk and 50% of the study period, respectively. FleXScan [32] was used to implement flexibly shaped spatial scan statistics, using a Poisson probability model, unrestricted log likelihood ratio, and flexible scanning method with a maximum spatial cluster size of 20 regions.

#### Spatiotemporal analyses of reporting sites

In order to assess the spatiotemporal distribution of PTB reporting facilities, which may confound spatiotemporal trends detected in PTB incidence, we geolocated PTB reporting facilities through application programming interfaces (APIs) provided by China’s top three digital mapping service providers: *Baidu*, *Tencent*, and *AutoNavi*. If the distance between any two or all three of the coordinates output by the APIs was less than 0.01°, the coordinates of the reporting facility were calculated as the centroid of these pairs or triplets. Otherwise, the facility name was manually entered into various search engines to extract its detailed address or, when necessary, that of a nearby landmark. These addresses were fed into the APIs again. For a given facility, if the distance between any two of the three coordinates returned by the APIs was still greater than 0.01°, the aforementioned process was repeated until the distance between at least one pair of the coordinates was less than 0.01°. The dates of operation of each PTB reporting facility were considered to extend from the first date of diagnosis referenced to the facility until end of the study period.

## Results

### Overall incidence rate

**Fig. 2:**
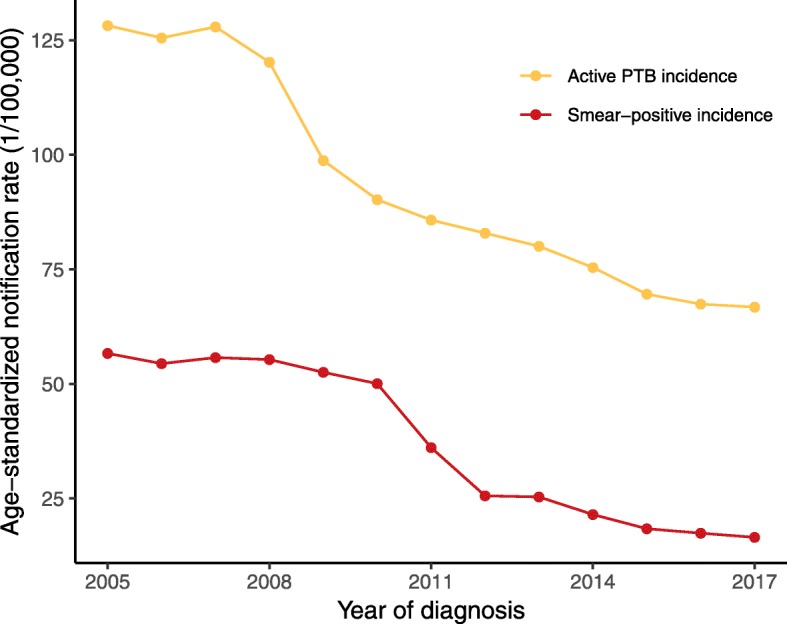
Age-standardized notification rate of reported active and smear-positive PTB cases in Sichuan Province, 2005–2017. PTB, pulmonary tuberculosis

A total of 976,873 active PTB cases were reported in the study area from 2005 to 2017, of which 388,739 (39.8%) were smear-positive cases. The annual mean direct age-standardized notification rates of active and smear-positive PTB cases were 93.7 and 37.3 per 100,000 persons, respectively. The notification rate of active PTB remained around 125 cases per 100,000 from 2005 to 2007 but decreased steadily thereafter, reaching 66.8 cases per 100,000 in 2017 (Fig. 2). The incidence rate of active PTB was was found to be decreasing more rapidly in Sichuan (at a rate of 3.77 cases per 100,000 per year, 95% CI: 3.28–4.31) than in China as a whole (3.14 cases per 100,000 per year, 95% CI: 2.61–3.67 [9]). The notification rate of smear-positive cases exhibited a similar downward trend from around 56.7 cases per 100,000 in 2005 to 16.5 cases per 100,000 in 2017. The annual proportion of smear-positive cases among all active PTB cases was 44.1% in 2005, peaking at 55.5% in 2010, and then subsequently decreasing to 25.1% in 2017.

### Demographic features

**Fig. 3:**
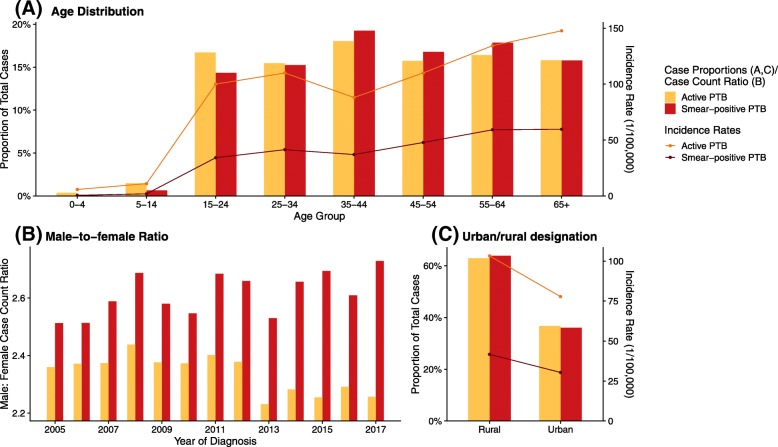
Demographic features of reported active and smear-positive PTB cases. (**a**): Age, (**b**): male-to-female ratio of cases; and (**c**): Distribution by urban/rural designation. PTB, pulmonary tuberculosis

The age distributions of active PTB cases (median 43, interquartile range (IQR) 29–59) and smear-positive PTB cases (median 45, IQR 31–59) were nearly identical (Fig. 3a). The median age of reported cases gradually increased for both active (0.51 year-old per year, 95% CI: 0.45–0.58) and smear-positive PTB (0.82 year-old per year, 95% CI: 0.75–0.90), but the rate of increase for smear-positive cases was significantly higher (Additional file 1: Figure S2). The rate of increase in median age for both active and smear-positive PTB cases was not significantly different between males and females. There was a similar proportion of active and smear-positive PTB cases among each age group, although there were fewer children and young adults among smear-positive cases than among active PTB cases. The reported incidence rates of active and smear-positive PTB were extremely low in age groups under 14, sharply increased for the 15–24 age group, and remained roughly constant in older groups, except for an unusually low incidence rate for the 35–44 age group.

A majority of reported PTB cases were male: the male-to-female case count ratio throughout the study period was 2.3:1 for active PTB cases, and 2.6:1 for smear-positive cases. The male-to-female case count ratio declined slightly over time for smear-positive PTB, but not for active PTB (Fig. 3b). For both active and smear-positive PTB, rural counties constituted a larger proportion of PTB cases, and exhibited a higher reported incidence rate (Fig. 3c). Farmers contributed the majority of active (66.1%) and smear-positive (71.6%) PTB cases, followed by students, unemployed individuals or those engaged in domestic household work, migrant workers, and laborers. However, since denominators for calculating occupation-specific reported incidence rate were not available, incidence rates could not be estimated by occupation (Additional file 1: Figure S3A). Incidence rates of PTB were highest in sparsely populated mountainous counties, though more cases were reported from the more densely populated, lower lying areas (Additional file 1: Figure S3B). Number and incidence of PTB in different demographic groups can be found in Additional file 1: Table S1.

### Seasonality

**Fig. 4:**
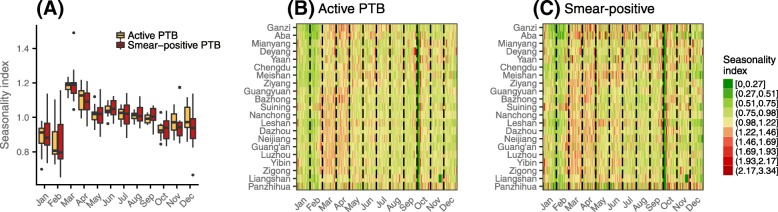
PTB seasonality. Seasonal distribution of (**a**) active and smear-positive PTB by month for the whole Sichuan; and seasonal distribution of reported (**b**) active and (**c**) smear-positive PTB cases for all 21 prefectures in Sichuan. The monthly (daily) seasonality index was calculated as the total number of reported cases in a certain month (calendar day) divided by the average monthly (daily) number of reported cases for that prefecture. The prefectures are ordered from top to bottom by increasing annual mean maximum temperature. PTB, pulmonary tuberculosis

Seasonal patterns of reported active and smear-positive PTB were similar. The ratio of observed case counts by month compared to the mean monthly case count for the year indicated that both active and smear-positive PTB cases were reported at an elevated rate in March when compared with the expected number of cases in the absence of any seasonal pattern (Fig. 4a). The first week of October exhibits an extremely low number of reported cases (Fig. 4b and c), despite higher than expected case counts in the previous and subsequent weeks; this phenomenon is likely due to a decrease in healthcare-seeking behavior and closure of outpatient facilities during a nationwide holiday that is usually observed from Oct 1st to 7th, and compensatory healthcare seeking in the immediate preceding and subsequent periods. Similar drops in the number of reported cases were also observed for other holidays.

The seasonal pattern of PTB varies by prefecture and shows some association with temperature (Fig. 4b and c). For example, the warmest two prefectures, Panzhihua and Liangshan, exhibit relatively weak seasonality, whereas the peak times of the number of reported cases for the coldest two prefectures, Ganzi and Aba, are around half a month later than those of other prefectures. However, since socioeconomic development, population density, landscape, and other climatic factors covary with temperature along the study region’s distinctive east-to-west gradient, a range of mechanisms may underlie these observations.

### Distribution of PTB reporting facilities

**Fig. 5:**
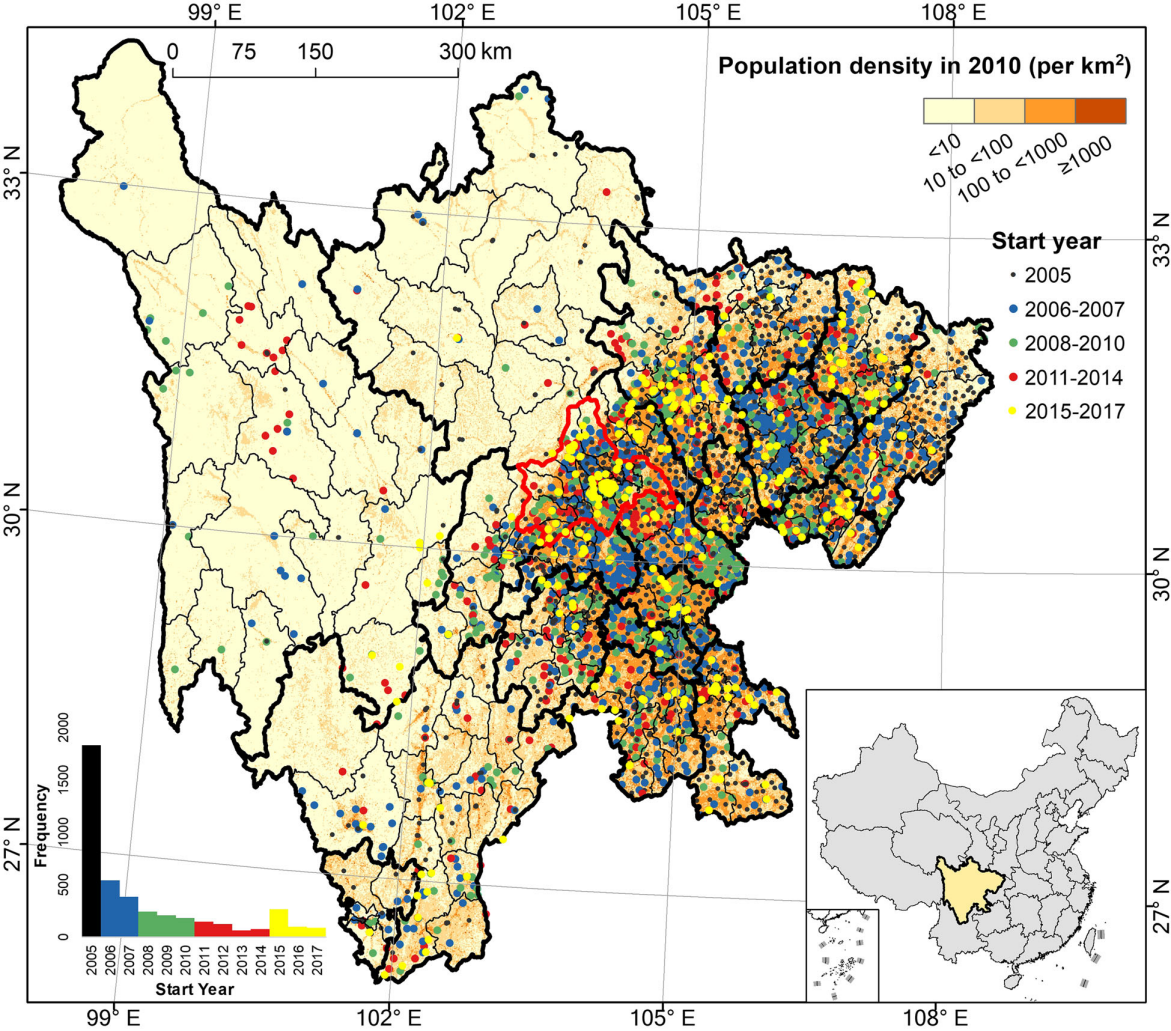
Geographic location of all PTB reporting facilities in Sichuan Province, China. Black, blue, green, red, and yellow points represent facilities that started reporting before and in 2005; in 2006–7; 2008–10; 2011–14; and 2015–17, respectively. Background color represents population density in 2010 [33], and heavy and light black lines represent prefecture and county boundaries, respectively. The boundary of Chengdu Prefecture, containing Sichuan’s capital, is delineated in red. Inset histogram shows the count of facilities that began reporting each year. Publically available administrative boundaries used in this visualization were obtained from China’s National Earth System Science Data Sharing Infrastructure [24]

To examine the spatial distribution of, and temporal trend in, PTB cases while controlling for the effect of changes in reporting effort, we geolocated and enumerated PTB reporting facilities in Sichuan, using this variable as a proxy for changing reporting. The spatial distribution of PTB reporting facilities in the study region followed the population distribution of the province, with high population and PTB reporting in the basin on the east side of the region (Fig. 5, inset map). More than 1,700 facilities began reporting PTB cases to NIDRS before or during 2005, with new reporting sites steadily added in the province each year (Fig. 5, inset histogram). Although some PTB reporting sites were established in less-populated and relatively undeveloped western counties between 2006 and 2014, the majority of reporting sites added between 2015 and 2017 were located in densely populated counties in the eastern region of the study area (Fig. 5, map).

### Geographic distribution

**Fig. 6:**
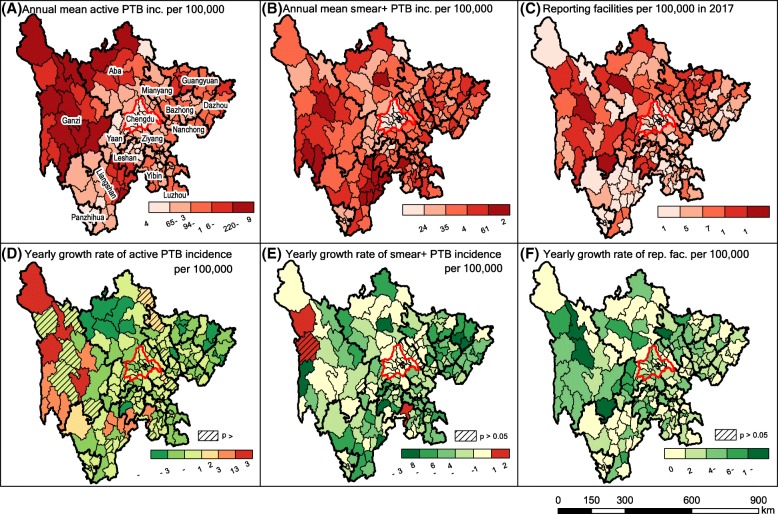
Spatial distributions and trends of PTB cases and reporting facilities from 2005 to 2017. Panels show annual mean reported (**a**) active and (**b**) smear-positive PTB incidence rate by county (prefectures, denoted by heavy border lines, are labeled with names in panel **a**); (**c**) number of reporting facilities per 100,000 persons in 2017; yearly growth rate of (**d**) active and (**e**) smear-positive PTB incidence estimated by linear regression from 2005 to 2017; and (**f**) yearly growth rate of number of reporting facilities per 100,000 persons from 2005 to 2017. Light lines represent county boundaries. Chengdu, the capital city, is delineated in red. PTB, pulmonary tuberculosis; Smear+, smear-positive; inc., incidence; rep. Fac., reporting facility. Publically available administrative boundaries used in this visualization were obtained from China’s National Earth System Science Data Sharing Infrastructure [24]

County mean annual incidence rates of reported active PTB ranged from 42.2 to 357.8 cases per 100,000. The median mean annual incidence rate across all counties was 90.5 cases per 100,000, with an IQR of 73.1–121.4. Chengdu and Panzhihua—prefectures with the two-highest gross regional products per capita in 2016 [20]—had the lowest mean reported active PTB incidence rates, while Ganzi and Aba, both prefectures in the mountainous areas of the Tibetan Plateau, had the highest incidence rates (Fig. 6a). However, despite having the highest annual mean incidence rate, counties in Aba prefecture exhibited sharp decreases in incidence from 2005 to 2017 (Fig. 6d). With the exception of Ganzi prefecture and an area in northeastern Liangshan prefecture, all counties experienced decreasing rates of active PTB during the study period (Fig. 6d). Both Ganzi and Liangshan are regions with limited economic development, poor access to healthcare, and populations with high proportions of ethnic minorities; northeast Liangshan also exhibits a high HIV incidence rate (Additional file 1: Figure S4). Increases in active PTB incidence were positively correlated with both reported HIV/AIDS incidence (Spearman’s rho = 0.16, *p*-value = 0.04) and proportion of ethnic minorities (Spearman’s rho = 0.26, p-value < 0.001).

The median mean annual incidence rate of reported smear-positive PTB cases was 38.0 cases per 100,000 (IQR 31.7–44.4, range 11.3–101.6). The spatial distribution of smear-positive PTB cases differed from that of active PTB cases: although Chengdu and Panzhihua still exhibited the lowest incidence rates, the highest incidence rate occurred in the east part of Liangshan (Fig. 6b). Incidence rates in most counties decreased, with the exception of significant increases in Dege *Xian* in Ganzi prefecture (increased by 0.96 cases per 100,000 persons per year), Yibin *Xian* in Yibin prefecture (0.92 cases per 100,000 persons per year), and Qingyang *Qu* in Chengdu prefecture (0.23 cases per 100,000 persons per year) (Fig. 6e). The Spearman correlation coefficient between counties’ mean annual reported smear-positive PTB incidence rates and their corresponding rates of change in incidence was − 0.40 (*p*-value < 0.001), suggesting that incidence rates have decreased faster in counties with higher baseline levels of PTB transmission. Increases in the PTB incidence were associated with both reported HIV/AIDS incidence (Spearman’s rho = 0.14, p-value = 0.06) and proportion of ethnic minorities (Spearman’s rho = 0.20, p-value = 0.007).

Surprisingly, the numbers of reporting facilities per 100,000 residents in Chengdu and Panzhihua were relatively low compared to other counties, whereas less populated and relatively undeveloped areas have higher reporting facility densities (Fig. 6c). Acknowledging that different tiers of healthcare facilities are distributed heterogeneously among rural and urban areas, while rural areas may have higher coverage of reporting facilities per unit population, facilities in these settings tend to be dominated by low-capacity township health centers. Such centers made up 41.8% of all reporting facilities 2005 to 2017 but only contributed 5.9% of all reported PTB cases. Ganzi prefecture had the highest increase in reporting facility density per 100,000 residents (Fig. 6f), even as the increase in absolute number of reporting facilities in this prefecture was lower than that observed for densely populated prefectures. No significant correlation was observed between the growth rates of both active and smear-positive PTB incidence rates and reporting facility density per 100,000 persons (p-value = 0.18 and 0.31, respectively, Additional file 1: Figure S5), suggesting that increases in incidence rates may be independent of the number of reporting facilities per 100,000 residents.

### Spatiotemporal cluster analyses

**Fig. 7:**
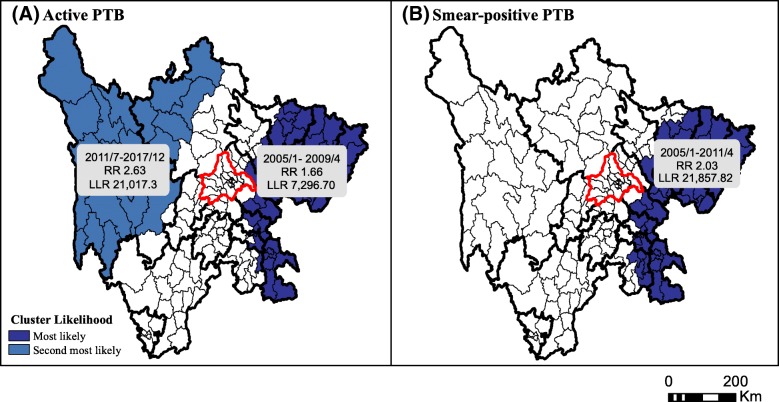
Top 5 significant (**a**) active and (**b**) smear-positive PTB clusters detected by SaTScan. RR represents the ratio of the risk of being a case in counties inside a cluster to the risk of that in counties in all other areas. LLR is log-likelihood ratio, and clusters were colored by decreasing LLR. Light and heavy lines represent county and prefecture boundaries, respectively. Chengdu, the capital city, is delineated in red. PTB, pulmonary tuberculosis. Publically available administrative boundaries used in this visualization were obtained from China’s National Earth System Science Data Sharing Infrastructure [24]

Both elliptic space-time scan statistics and flexibly shaped scan statistics revealed clusters of elevated active TB incidence in the eastern and northwestern areas of the study region (Fig. 7 and Additional file 1: Figure S6). Number of observed and expected cases are presented in Additional file 1: Tables S2–S5. The eastern region of the province was identified as the most likely cluster for both active and smear positive PTB. The northwestern region, including Ganzi prefecture and the western Aba prefecture, was identified as an emerging active PTB cluster, beginning in July, 2011 and persisting to the end of the study period.

## Discussion

We investigated demographic and spatiotemporal trends in active and smear-positive PTB cases across 13 years of passive surveillance data in a region with a large population-at-risk. We found that the overall reported incidence rate of PTB has decreased in the region, but that there is both spatial and temporal heterogeneity in incidence rates across counties over the study period. We identified persistent clusters of ongoing transmission, including counties where the incidence rate is increasing, particularly in areas with high HIV incidence and those with substantial ethnic minority populations (Additional file 1: Figure S4). The clusters were generally consistent with the results of previous research [11, 12], and may be explained by the limited access to healthcare or presence of major transportation hubs in these regions. We observed modest seasonality for PTB in the study region during the study period, with more cases being reported in March and April, and fewer cases in January and February.

Our results indicated that the incidence rate of PTB was extremely low in those age < 14, and that the median age of reported PTB cases was increasing, suggesting that the current TB prevention and control measures in China are generally succeeding in the region. The Bacillus Calmette–Guérin (BCG) vaccine has been mandatory in China since 1954 [34, 35], and the proportion of patients detected at hospitals undergoing treatment in TB control institutions increased from 63.6% in 2006 to 93.5% in 2010 [36]. Further evidence suggesting that the transmission rate of PTB has decreased over the past decade comes from comparing our results to other findings: a 2008 study examining the incidence of PTB among 5-year age groups from 2000 to 2006 found the highest incidence for the 20–24, 40–45, and 65–74 year-old groups [10], while the age groups with the highest incidence for our study period were the 25–30, 50–55, and 70–75 year old groups.

We observed modest seasonal trends for PTB, with a higher number of cases in the spring and summer, which is consistent with patterns observed elsewhere in the world. High numbers of PTB cases in March and April have been reported throughout the northern hemisphere, including in other regions of China [37–39], Mongolia [40], the United States [41], and Peru [42], but at the same time there are exceptions to these patterns [43–46]. Indeed, the drivers of the seasonality of PTB remain poorly understood. Given the long time period between the exposure and the onset of TB, one possible explanation for the excessive number of reported cases in spring and summer is increased transmission during winter, because both low temperature and high PM_2.5_ level may increase the time spent indoors with poor ventilation [39, 47, 48]. Another possible explanation is the weakened host immunity during winter, which is associated with the high prevalence of other respiratory infectious diseases, vitamin D deficiency due to insufficient ultraviolet radiation exposure, and high PM_2.5_ level, which can impair respiratory system immune response [17, 39, 48].

The spatial distribution of annual mean incidence rates of reported active PTB cases reported here is generally consistent with the results of previous research [11, 12]. Clusters of both active and smear-positive PTB were detected in eastern Sichuan, possible owing to its early economic stage of development (see Additional file 1: Figure S7 for county-level GDP per capita in 2016), limited access to healthcare in the region, and the presence of major transportation hubs, which are associated with increased contact rates between travelers and local residents [49, 50]. An emerging cluster of active PTB was found in the Ganzi and western Aba prefectures from 2011 to 2017, which are also exhibiting increasing growth rates of reported PTB incidence rates. These prefectures are also at an early stage of economic development (see Additional file 1: Figure S7), and importantly, ethnic minorities in these regions—especially Tibetan people—may be at higher risk of active PTB as compared with other ethnic groups, possibly due to genetic susceptibility [51], nomadic lifestyle [52], and particularly low socioeconomic status [53]. This emerging cluster was detectable among active cases, but not smear-positive, which might be a consequence of the low laboratory capacity in these areas.

Among the important limitations of this study, the reliance on a passive surveillance system can yield estimated incidence rates that may be affected by individuals’ healthcare seeking behavior, diagnostic performance, and facility compliance, and key reporting biases may differ across demographic groups or counties. We attempted to control for variable reporting effort by using reporting facility density as a proxy variable, and found no relation with changes in PTB incidence. However, this approach has significant limitations in the assumption that all reporting facilities perform equally. Another challenge in relying on NIDRS data arises from the reporting guidelines and procedures, which have evolved substantially since the system was initiated, leading to possible inconsistencies in case definitions, reporting infrastructures, and data accuracy over time. To mitigate the influence of dramatically increasing case detection rate on the results, we excluded the data for 2004, the year when NIDRS was established, from the analyses. According to WHO’s estimation, case detection rate of TB increased 10% (64% to 74%) from 2003 to 2004 alone, and another 13% from 2005 to 2017 [46]. Finally, compared to the diagnostic methods of many acute infectious diseases, diagnostic methods for PTB can be ambiguous and inconclusive [1], which can contribute bias associated with misclassification of outcome. Although suspected cases were excluded in the analyses, misdiagnosis and under-ascertainment may still exist.

## Conclusions

The present study suggests that the overall reported incidence rate of PTB is decreasing at the province-level, but progress has not been equally distributed. Achieving the goal of reducing the incidence rate to below 58 per 100,000 people by 2020 [54] will require additional resources and control efforts directed at western Sichuan, where HIV prevalence is high, the reported incidence rate of PTB is increasing, and the laboratory capacity is low. Future research should focus on achieving a greater understanding of the environmental and socioeconomic drivers of PTB in this region—including temperature, sunlight exposure, PM_2.5_ level, GDP, prevalence of other respiratory infectious diseases and HIV/AIDS—in order to better understand the observed spatiotemporal pattern and to inform targeted interventions.

## Additional files

Additional file 1: **Figure S1.** Daily mean case count aggregated by date of onset from 2005 to 2017. **Figure S2.** (A) Median and (B) mean age at diagnosis for active and smear-positive PTB, and their linear trends. **Figure S3.** Demographic features of reported active and smear-positive PTB cases. **Figure S4.** (A) County-level average annual reported HIV and AIDS incidence rate (1/100,000) from 2005 to 2017. (B) Proportion of ethnic minority for each county. **Figure S5.** Relationship between yearly growth rate of reporting facility density and yearly growth rate of (A) active and (B) smear-positive PTB (1/100,000 per year). **Figure S6.** Top 5 significant (A) active and (B) smear-positive PTB clusters detected by FlexScan. **Figure S7.** GDP per capita for each county in 2016. Source: Sichuan Statistical Yearbook 2017. **Table S1**. Characteristics of active and smear positive PTB cases in Sichuan province, 2005–2017. **Table S2.** Active PTB clusters from SaTScan. **Table S3.** Top 5 active PTB clusters from FleXScan. **Table S4.** Smear positive PTB clusters from SaTScan. **Table S5.** Top 5 smear positive PTB clusters from FleXScan. (DOCX 1833 kb)

## Data Availability

The datasets generated and/or analysed are not publicly available due to the protection of personal information, but are available from the corresponding author on reasonable request.

## Abbreviations

API: Application programming interface
BCG: Bacillus Calmette–Guérin
CI: Confidence interval
GDP: Gross domestic product
IQR: Interquartile range
LOESS: Locally Weighted Regression
NIDRS: National Infectious Disease Reporting System
PTB: Pulmonary tuberculosis
STL: Seasonal-trend decomposition procedure based on LOESS
TB: Tuberculosis

## References

[1] Campbell IA, Bah-Sow O (2006). Pulmonary tuberculosis: diagnosis and treatment. BMJ: British Medical Journal.

[2] World Health Organization: Global tuberculosis report 2018: World Health Organization; 2018.

[3] Beavers SF, Pascopella L, Davidow AL, Mangan JM, Hirsch-Moverman YR, Golub JE, Blumberg HM, Webb RM, Royce RA, Buskin SE (2018). Tuberculosis mortality in the United States: epidemiology and prevention opportunities. Ann Am Thorac Soc.

[4] Li X-X, Wang L-X, Zhang J, Liu Y-X, Zhang H, Jiang S-W, Chen J-X, Zhou X-N (2014). Exploration of ecological factors related to the spatial heterogeneity of tuberculosis prevalence in P. R China. Global Health Action.

[5] Sun W, Gong J, Zhou J, Zhao Y, Tan J, Ibrahim AN, Zhou Y (2015). A spatial, social and environmental study of tuberculosis in China using statistical and GIS technology. Int J Environ Res Public Health.

[6] Zhao F, Cheng S, He G, Huang F, Zhang H, Xu B, Murimwa TC, Cheng J, Hu D, Wang L (2013). Space-time clustering characteristics of tuberculosis in China, 2005-2011. PLoS One.

[7] Li T, He J, Zhang P, Xia Y, Wang D, Li Y, Wu J. Epidemiology of Tuberculosis in Sichuan in 2012. J Prev Med Inf. 2013;29(11).

[8] Wang L, Zhang H, Ruan Y, Chin DP, Xia Y, Cheng S, Chen M, Zhao Y, Jiang S, Du X (2014). Tuberculosis prevalence in China, 1990–2010; a longitudinal analysis of national survey data. Lancet.

[9] National Health and Family Planning Commission (2017). China’s Health And Family Planning Statistical Yearbook 2017.

[10] Yang X-y, Zhang N-m, Diao X, Mao X, Li Y-P (2008). Epidemiological analysis of pulmonary tuberculosis in Sichuan Province, China, 2000–2006. Int J Infect Dis.

[11] Li T, He J, Yang C, Li Y, Wang D, Chen C, Wu J (2016). A clustering study on pulmonary tuberculosis based on pure spatial clustering and spatiotemporal scanning. Chinese J Antituberculosis.

[12] Li T, Yang CH, He JG, Li YK, Xiao Y, Li J, Wang DX, Chen C, Wu JL (2017). Spatial-temporal distribution of smear positive pulmonary tuberculosis in Liangshan Yi autonomous prefecture, Sichuan province, 2011-2016. Zhonghua liu xing bing xue za zhi= Zhonghua liuxingbingxue zazhi.

[13] National Bureau of Statistics of China (2012). Tabulation on the 2010 population census of the People’s Republic of China.

[14] Permanent residents in Sichuan reached 83.02 million [http://www.sc.gov.cn/10462/10464/10797/2018/4/6/10448426.shtml].

[15] Baker M, Das D, Venugopal K, Howden-Chapman P (2008). Tuberculosis associated with household crowding in a developed country. J Epidemiol Community Health.

[16] Cegielski JP, McMurray DN: The relationship between malnutrition and tuberculosis: evidence from studies in humans and experimental animals.13. 15139466

[17] Koh GCKW, Hawthorne G, Turner AM, Kunst H, Dedicoat M (2013). Tuberculosis incidence correlates with sunshine: an ecological 28-year time series study. PLoS One.

[18] Xiao Y, He L, Chen Y, Wang Q, Meng Q, Chang W, Xiong L, Yu Z (2018). The influence of meteorological factors on tuberculosis incidence in Southwest China from 2006 to 2015. Sci Rep.

[19] Liang S, Yang C, Zhong B, Guo J, Li H, Carlton EJ, Freeman MC, Remais JV (2014). Surveillance systems for neglected tropical diseases: global lessons from China’s evolving schistosomiasis reporting systems, 1949–2014. Emerg Themes Epidemiol.

[20] Sichuan Statistical Bureau (2017). Sichuan Statistical Yearbook 2006-2017.

[21] National Bureau of Statistics of China (2012). Tabulation on the 2010 population census of the People’s Republic of China by nationality.

[22] National Bureau of statistics of China (2011). China county-level economy yearbook 2010.

[23] Abatzoglou JT, Dobrowski SZ, Parks SA, Hegewisch KC (2018). TerraClimate, a high-resolution global dataset of monthly climate and climatic water balance from 1958–2015. Scientific Data.

[24] National Earth System Science Data Sharing Infrastructure [http://www.geodata.cn/].

[25] Ministry of Health P. R. China: Diagnostic Criteria for Pulmonary Tuberculosis. In*.*: People’s Medical Publishing House; 2008.

[26] Li XX, Wang LX, Zhang H, Du X, Jiang SW, Shen T, Zhang YP, Zeng G (2013). Seasonal variations in notification of active tuberculosis cases in China, 2005-2012. PLoS One.

[27] Cleveland R, Cleveland W, McRae J, Terpenning I (1990). STL: a seasonal-trend decomposition. J Off Stat.

[28] R Core Team: R: a language and environment for statistical computing. Vienna, Austria: R Foundation for statistical Computing; 2017.

[29] Kulldorff M, Huang L, Pickle L, Duczmal L (2006). An elliptic spatial scan statistic. Stat Med.

[30] Tango T, Takahashi K (2005). A flexibly shaped spatial scan statistic for detecting clusters. Int J Health Geogr.

[31] Kulldorff M, Heffernan R, Hartman J, Assunção R, Mostashari F (2005). A space–time permutation scan statistic for disease outbreak detection. PLoS Med.

[32] FleXScan: Software for the Flexible Scan Statistics.

[33] Dobson JE, Bright EA, Coleman PR, Durfee RC, Worley BA (2000). LandScan: a global population database for estimating populations at risk. Photogramm Eng Remote Sens.

[34] Trunz BB, Fine PEM, Dye C (2006). Effect of BCG vaccination on childhood tuberculous meningitis and miliary tuberculosis worldwide: a meta-analysis and assessment of cost-effectiveness. Lancet.

[35] Zheng Y, Rodewald L, Yang J, Qin Y, Pang M, Feng L, Yu H (2018). The landscape of vaccines in China: history, classification, supply, and price. BMC Infect Dis.

[36] Song Y, Xia Y (2012). Referral tracking of TB patients reported by Sichuan non-TBControl institutions on network, 2006-2011. J Prev Med Inf.

[37] Wubuli A, Li Y, Xue F, Yao X, Upur H, Wushouer Q (2017). Seasonality of active tuberculosis notification from 2005 to 2014 in Xinjiang, China. PLoS One.

[38] Yang X, Duan Q, Wang J, Zhang Z, Jiang G (2014). Seasonal variation of newly notified pulmonary tuberculosis cases from 2004 to 2013 in Wuhan, China. PLoS One.

[39] You S, Tong YW, Neoh KG, Dai Y, Wang CH (2016). On the association between outdoor PM 2.5 concentration and the seasonality of tuberculosis for Beijing and Hong Kong ☆. Environ Pollut.

[40] Naranbat N, Nymadawa P, Schopfer K, Rieder H (2009). Seasonality of tuberculosis in an eastern-Asian country with an extreme continental climate. Eur Respir J.

[41] Willis MD, Winston CA, Heilig CM, Cain KP, Walter ND, Mac Kenzie WR (2012). Seasonality of tuberculosis in the United States, 1993-2008. Clin Infect Dis.

[42] Tom W, Schumacher SG, Gurjinder S, Tovar MA, Karine Z, Baldwin MR, Rosario M, Ramos ES, Chulanee J, Lewis JJ (2014). The seasonality of tuberculosis, sunlight, vitamin D, and household crowding. J Infect Dis.

[43] Douglas AS, Strachan DP, Maxwell JD (1996). Seasonality of tuberculosis: the reverse of other respiratory diseases in the UK. Thorax.

[44] Leung CC, Yew WW, Chan TY, Tam CM, Chan CY, Chan CK, Tang N, Chang KC, Law WS (2005). Seasonal pattern of tuberculosis in Hong Kong. Int J Epidemiol.

[45] Wah W, Das S, Earnest A, Lim LKY, Chee CBE, Cook AR, Wang YT, Win KMK, Ong MEH, Li YH (2014). Time series analysis of demographic and temporal trends of tuberculosis in Singapore. BMC Public Health.

[46] WHO Tuberculosis data download [https://www.who.int/tb/country/data/download/en/].

[47] Tedijanto C, Hermans S, Cobelens F, Wood R, Andrews JR (2018). Drivers of seasonal variation in tuberculosis incidence: insights from a systematic review and mathematical model. Epidemiology.

[48] Guo C, Du Y, Shen SQ, Lao XQ, Qian J, Ou CQ (2017). Spatiotemporal analysis of tuberculosis incidence and its associated factors in mainland China. Epidemiol Infect.

[49] Kenyon TA, Valway SE, Ihle WW, Onorato IM, Castro KG (1996). Transmission of multidrug-resistant mycobacterium tuberculosis during a long airplane flight. N Engl J Med.

[50] Liu J, Yao H, Liu E (2005). Analysis of factors affecting the epidemiology of tuberculosis in China. Int J Tuberc Lung Dis.

[51] Zhou WJ, Hu XJ, Zhang JY, Zhou Y, Wu LJ, Wang MJ, Wang N, Lu XJ, Ying BW (2016). Association of Gene Polymorphisms in Wnt Signal Pathway with Tuberculosis in Chinese Tibetan Population. Sichuan da xue xue bao Yi xue ban.

[52] Wang CC, Liu Y, Chao XZ, Jiang MX, Er-Chen LI: Epidemiological characteristics of pulmonary tuberculosis of Qinghai,2006-2015. Modern Preventive Medicine 2017.

[53] Wang D, Rao Z (2015). Comparison of registered incidence of smear positive pulmonary tuberculosis patients in different nationalities in Sichuan Province. J Prev Med Inf.

[54] Notice of the General Office of the People’s Government of Sichuan Province on printing and distributing the “13th Five-Year Plan” Sichuan Province Tuberculosis Prevention and Control Plan [http://www.sc.gov.cn/10462/10464/13298/13301/2017/5/30/10424023.shtml].

