# Supplementary material for: Evidence for heterogeneity in China’s progress against pulmonary tuberculosis: uneven reductions in a major center of ongoing transmission, 2005–2017
Source: BMC Infect Dis. 2019 Jul 12;19:615. doi: 10.1186/s12879-019-4262-2 (PMC6626433; doi:10.1186/s12879-019-4262-2)
Supplement: Supplementary file 1 — Figure S1. Daily mean case count aggregated by date of onset from 2005 to 2017. Figure S2. (A) Median and (B) mean age at diagnosis for active and smear-positive PTB, and their linear trends. Figure S3. Demographic features of reported active and smear-positive PTB cases. Figure S4. (A) County-level average annual reported HIV and AIDS incidence rate (1/100,000) from 2005 to 2017. (B) Proportion of ethnic minority for each county. Figure S5. Relationship between yearly growth rate of reporting facility density and yearly growth rate of (A) active and (B) smear-positive PTB (1/100,000 per year). Figure S6. Top 5 significant (A) active and (B) smear-positive PTB clusters detected by FlexScan. Figure S7. GDP per capita for each county in 2016. Source: Sichuan Statistical Yearbook 2017. Table S1. Characteristics of active and smear positive PTB cases in Sichuan province, 2005–2017. Table S2. Active PTB clusters from SaTScan. Table S3. Top 5 active PTB clusters from FleXScan. Table S4. Smear positive PTB clusters from SaTScan. Table S5. Top 5 smear positive PTB clusters from FleXScan. (DOCX 1833 kb) [file 12879_2019_4262_MOESM1_ESM.docx]

## Additional File 1

**Supplementary methods**

Elliptic space-time scan procedure involves scanning a series of elliptic cylindrical windows with various sizes and angles of bottom surfaces, locations, and heights, which represent the spatial extents, centroids, and time periods of potential clusters over the study area. A log-likelihood ratio (LLR) representing the likelihood that individuals inside the current window have a higher probability of being a case than those outside the window, compared to the likelihood that all individuals have the same probability of being cases, is estimated under a probability model chosen according to the nature of the data. The relative risk (RR) of a detected cluster is estimated as the risk of being a case inside the cluster to that outside of the cluster, with inference obtained through Monte Carlo simulations [1]. The flexibly shaped spatial scan procedure involves, for each region *i*, investigating all sets of connected regions containing region *i* inside circles centered at *i*, which yields a substantial computational burden compared to circular space-time scan statistics [2].

## References

1. Kulldorff M. Prospective time periodic geographical disease surveillance using a scan statistic. J R Stat Soc Ser A Stat Soc **2001**; 164: 61–72. https://doi.org/10.1111/1467-985X.00186

2. Tango T, Takahashi K. A flexibly shaped spatial scan statistic for detecting clusters. Int J Health Geogr **2005**; 4: 11. <https://doi.org/10.1186/1476-072X-4-11>

**Figure S1.** Daily mean case count aggregated by date of onset from 2005 to 2017. Because of recall bias, the case count is disproportionately high for the 1st, 10th, and 20th days of each month.


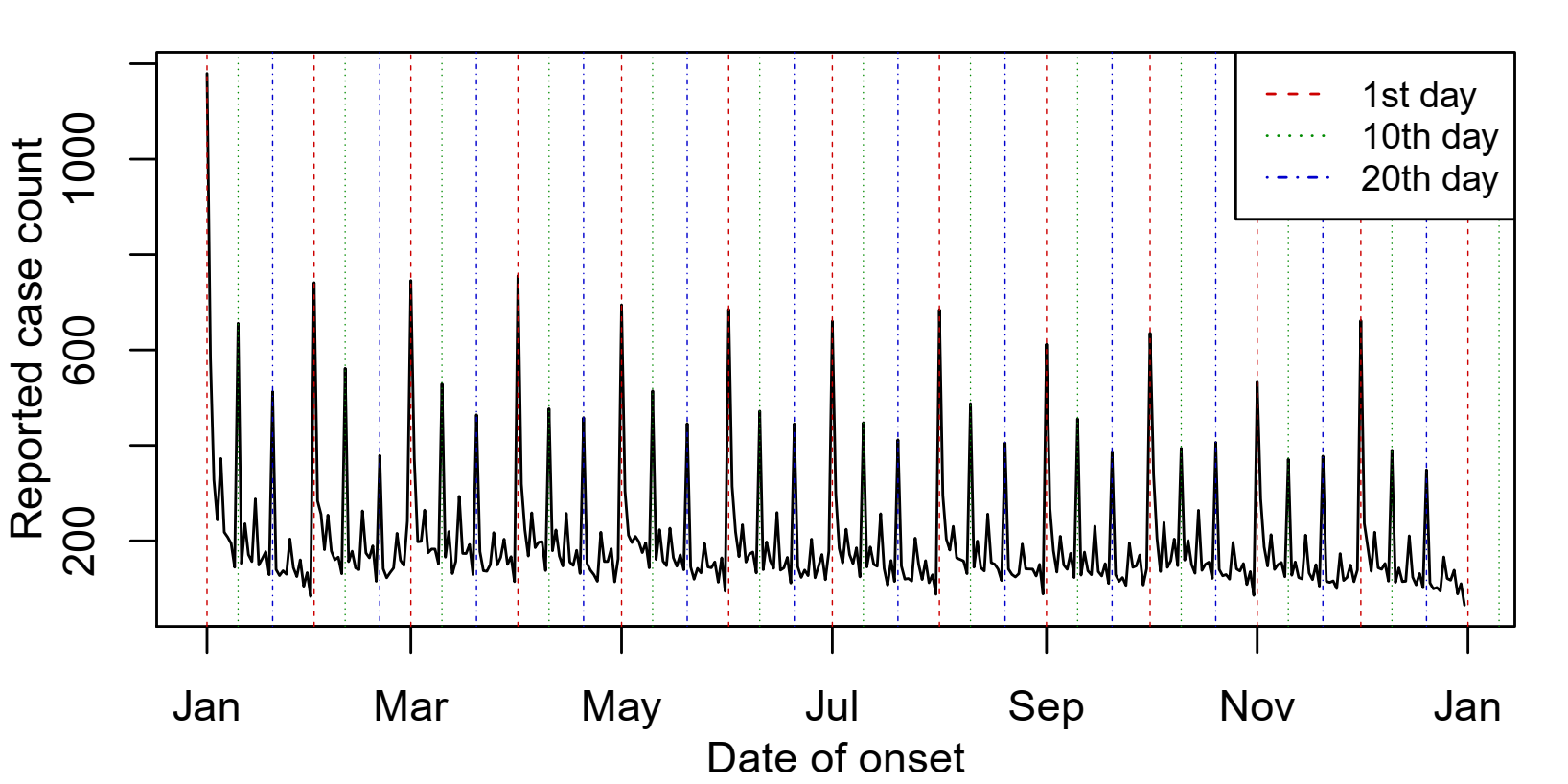


**Figure S2.** (A) Median and (B) mean age at diagnosis for active and smear-positive PTB, and their linear trends.


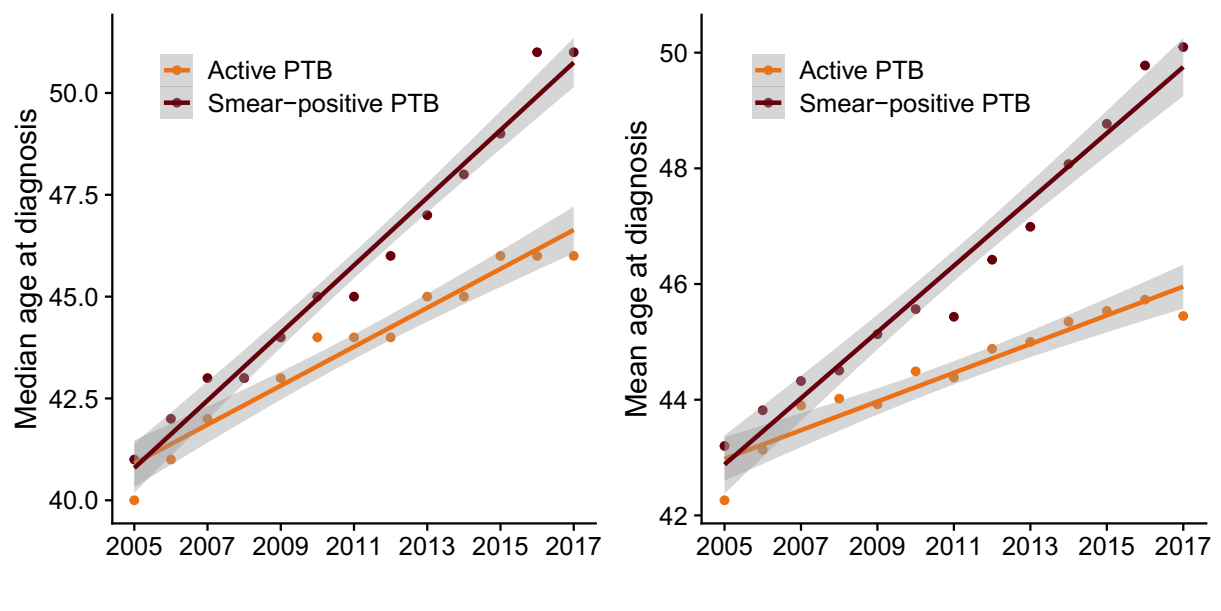


**Figure S3.** Demographic features of reported active and smear-positive PTB cases. (A): Occupation (“DW/None” refers to domestic workers or unemployed persons; “Migr. Worker” refers to migrant workers); (B): Distribution by predominant landform.


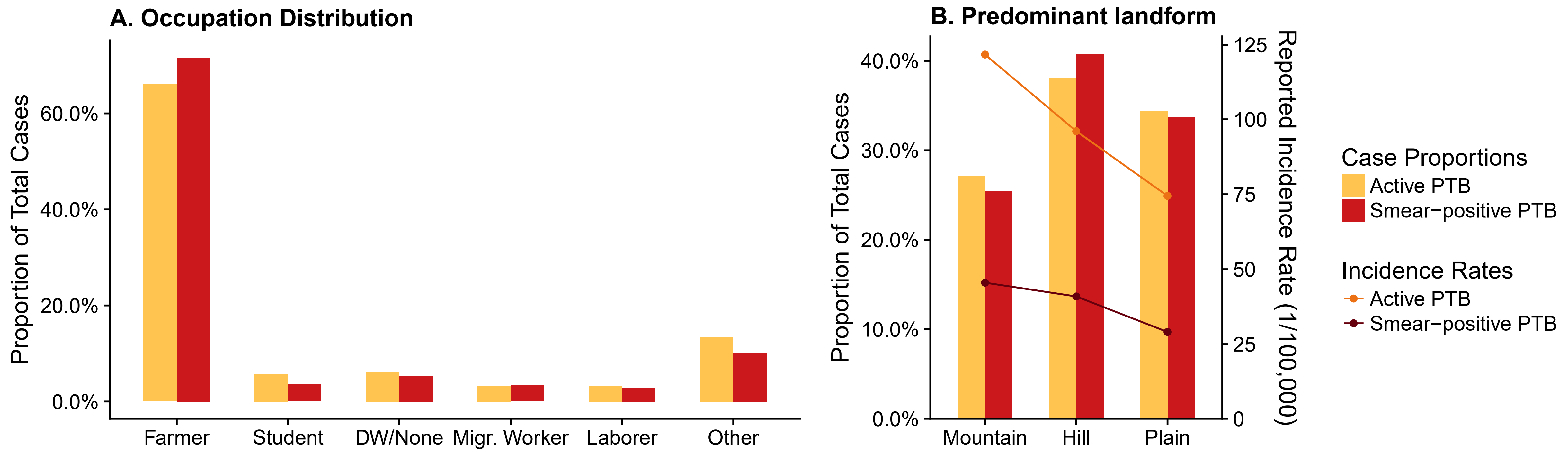


**Figure S4.** (A) County-level average annual reported HIV and AIDS incidence rate (1/100,000) from 2005 to 2017. (B) Proportion of ethnic minority for each county.


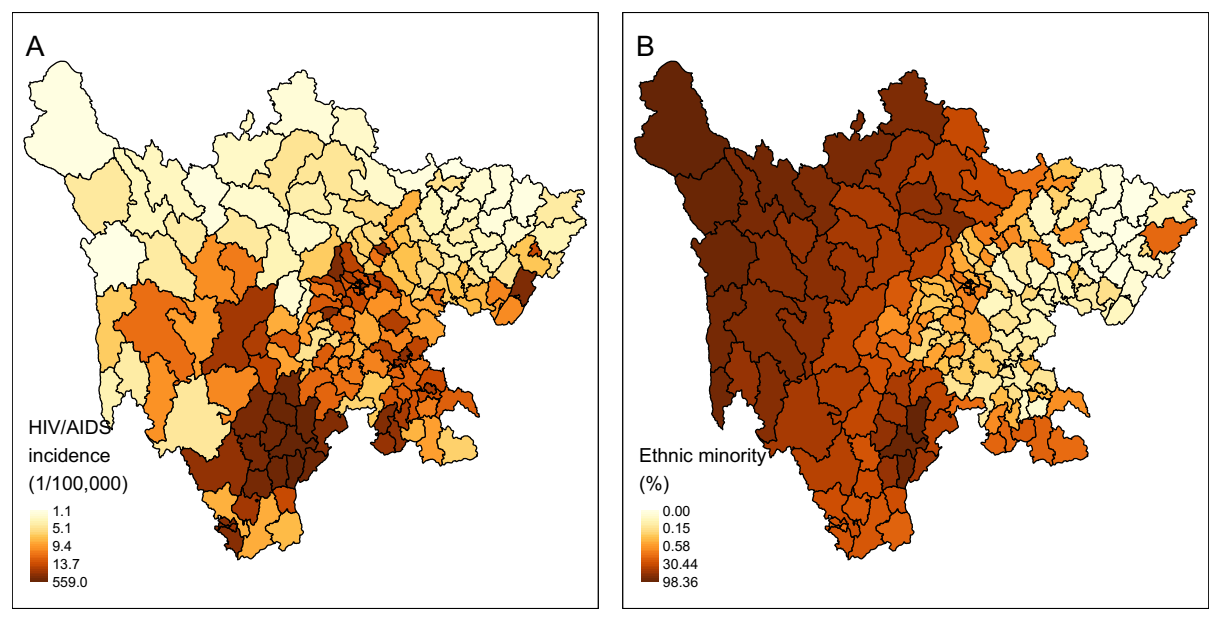


**Figure S5.** Relationship between yearly growth rate of reporting facility density and yearly growth rate of (A) active and (B) smear-positive PTB (1/100,000 per year).


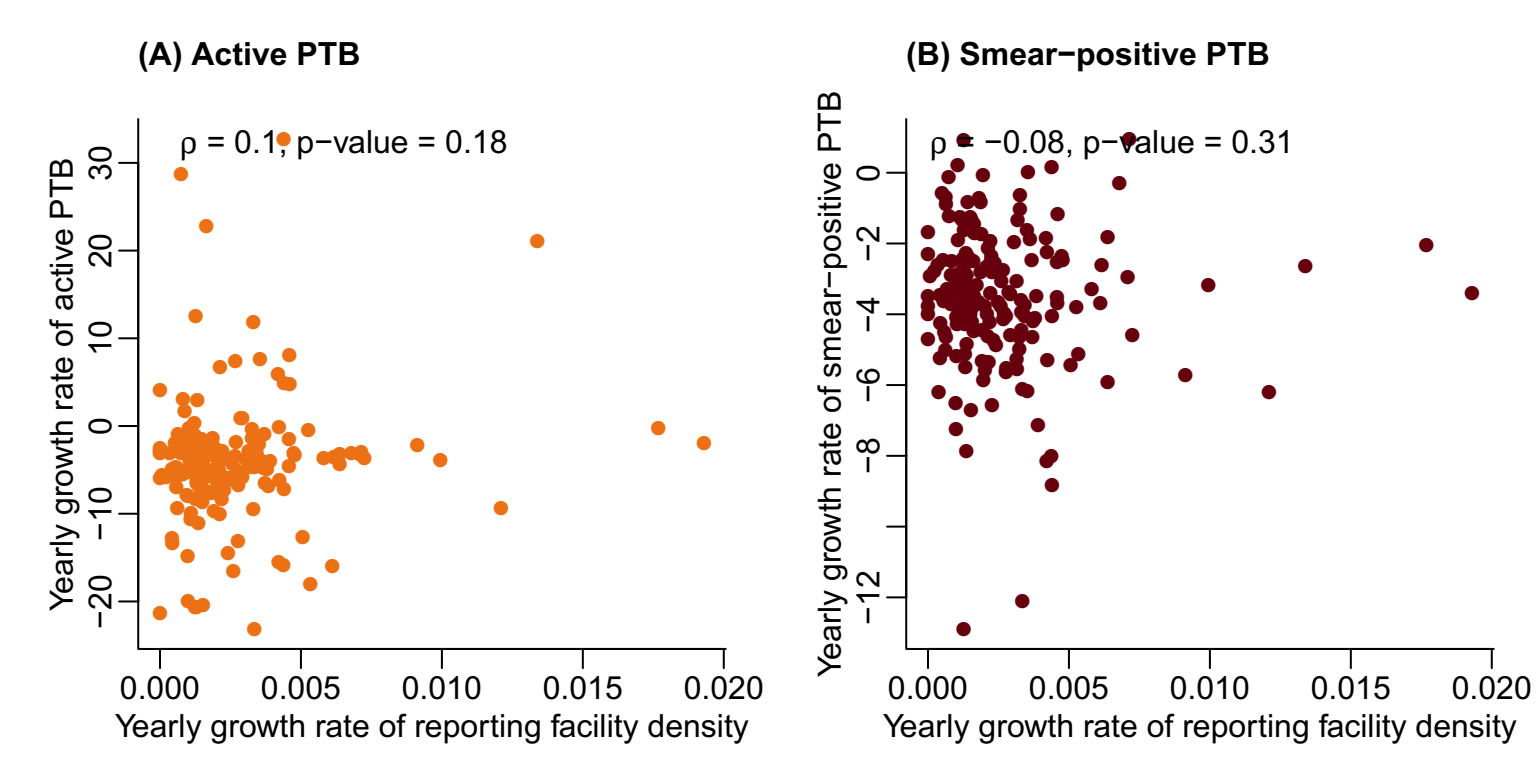


**Figure S6.** Top 5 significant (A) active and (B) smear-positive PTB clusters detected by FlexScan. RR represents the ratio of the risk of being a case in counties inside a cluster to the risk of that in counties in all other areas. LLR is log-likelihood ratio, and clusters are colored by decreasing LLR. Light and heavy lines represent county and prefecture boundaries, respectively. Chengdu, the capital city, is delineated in red.


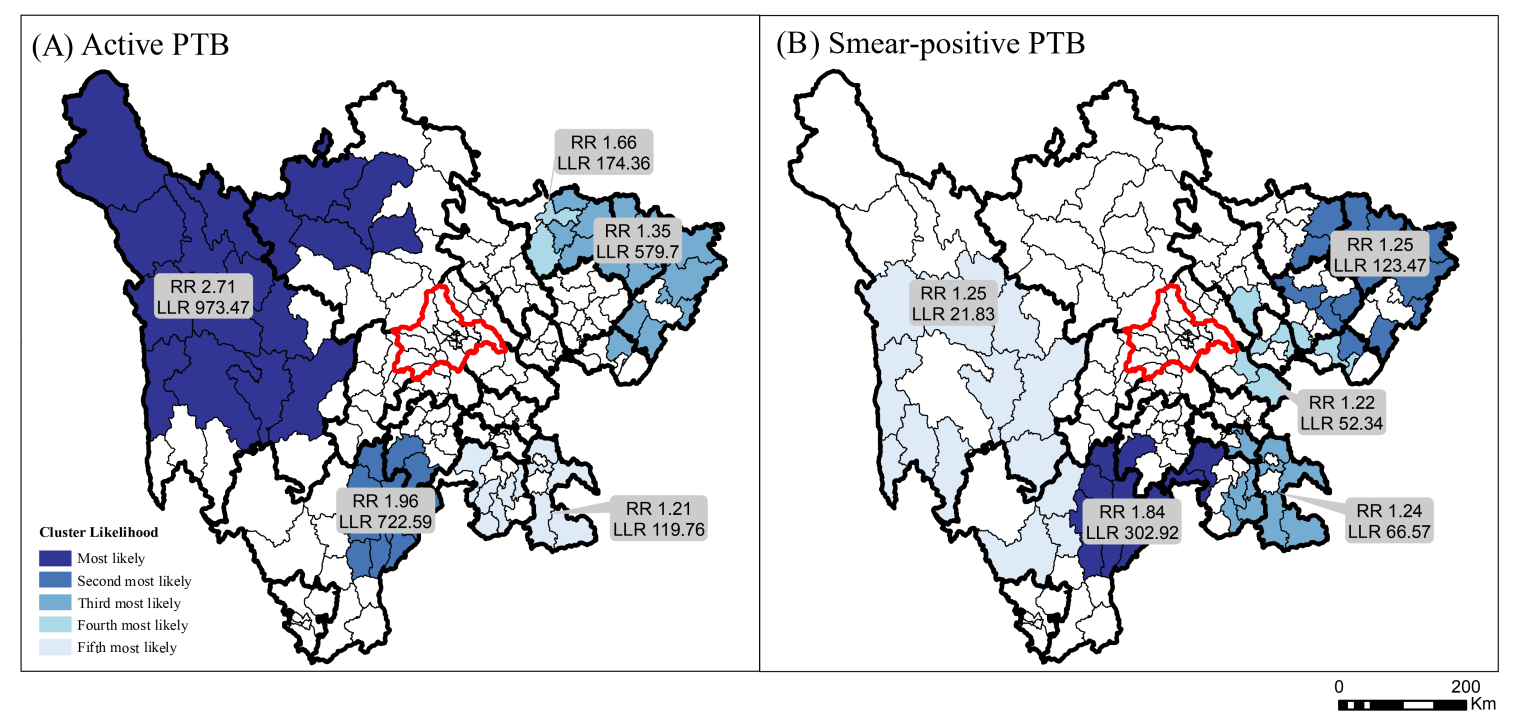


**Figure S7.** GDP per capita for each county in 2016. Source: Sichuan Statistical Yearbook 2017..


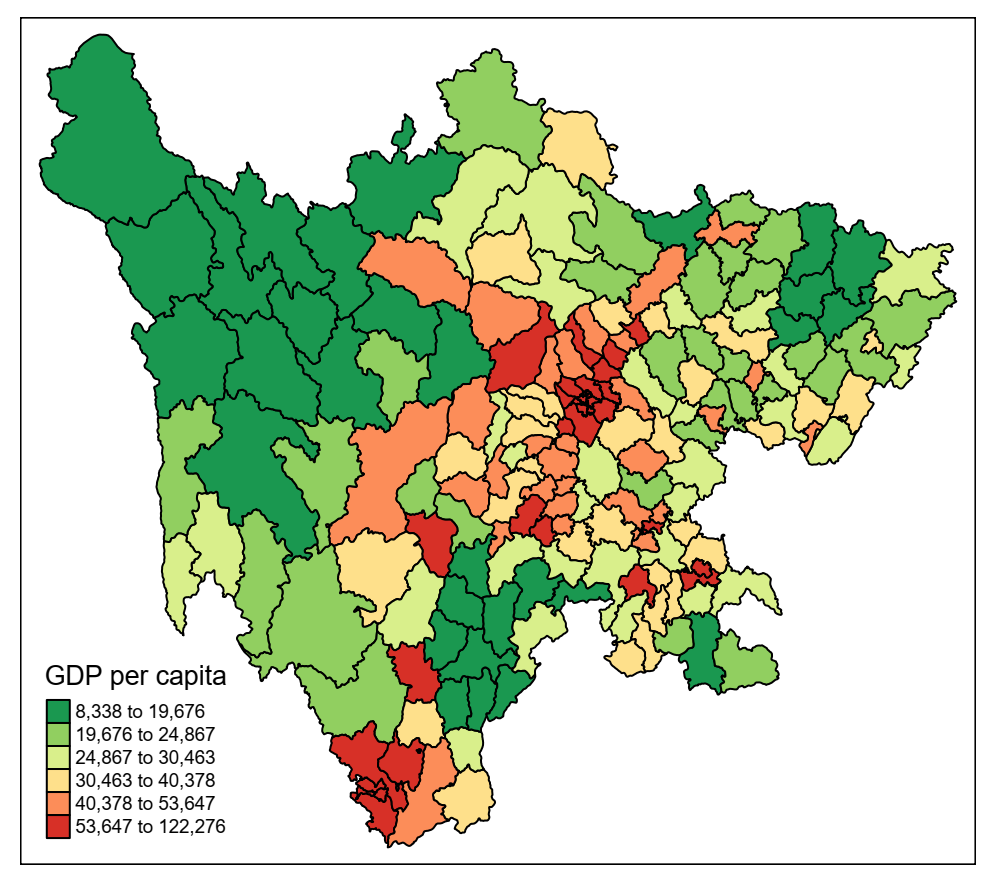


**Table S1**. Characteristics of active and smear positive PTB cases in Sichuan province, 2005–2017

| **Characteristics** | **Active PTB** (n=976873) | |  | **Smear positive PTB** (n=388739) | |
| --- | --- | --- | --- | --- | --- |
|  | Number of cases (%) | Incidence rate (/100000) |  | Number of cases (%) | Incidence rate (/100000) |
| **Gender** |  |  |  |  |  |
| Male | 684930 (70.11) | 131.75 |  | 280664 (72.20) | 53.99 |
| Female | 291943 (29.89) | 54.45 |  | 108075 (27.80) | 20.16 |
| **Age Group** |  |  |  |  |  |
| 0-4 | 3477 (0.36) | 5.78 |  | 337 (0.09) | 0.55 |
| 5-14 | 14500 (1.48) | 10.98 |  | 2534 (0.65) | 1.89 |
| 15-24 | 163243 (16.71) | 100.00 |  | 55736 (14.34) | 34.17 |
| 25-34 | 151030 (15.46) | 109.96 |  | 59243 (15.24) | 41.38 |
| 35-44 | 176325 (18.05) | 88.05 |  | 74798 (19.24) | 37.02 |
| 45-54 | 153715 (15.74) | 110.04 |  | 65262 (16.79) | 47.80 |
| 55-64 | 160204 (16.40) | 134.37 |  | 69448 (17.87) | 59.15 |
| 65+ | 154369 (15.80) | 147.73 |  | 61378 (15.79) | 59.62 |
| **Occupation** |  |  |  |  |  |
| Farmer | 645855 (66.11) | - |  | 278487 (71.64) | - |
| Students | 56280 (5.76) | - |  | 14288 (3.68) | - |
| Household/Domestic work or unemployed | 60370 (6.18) | - |  | 20621 (5.3) | - |
| Migrant worker | 31486 (3.22) | - |  | 13195 (3.39) | - |
| Worker | 31555 (3.23) | - |  | 11030 (2.84) | - |
| Others | 131100 (13.42) | - |  | 39365 (10.13) | - |
| NA | 20227 (2.07) | - |  | 11753 (3.02) | - |
| **Landform** |  |  |  |  |  |
| Mountain | 265133 (27.14) | 121.72 |  | 99034 (25.48) | 45.47 |
| Hill | 372046 (38.09) | 96.11 |  | 158214 (40.7) | 40.87 |
| Plain | 335927 (34.39) | 74.47 |  | 130811 (33.65) | 29.00 |
| NA | 3767 (0.39) | - |  | 680 (0.17) | - |
| **Urbanization** |  |  |  |  |  |
| Rural area | 614406 (62.9) | 103.28 |  | 248034 (63.8) | 41.69 |
| Urban area | 358700 (36.72) | 77.79 |  | 140025 (36.02) | 30.37 |
| NA | 3767 (0.39) | - |  | 680 (0.17) | - |

**Table S2.** Active PTB clusters from SaTScan

| **ID** | **LLR** | | **P value** | | **RR** | | **Observed Cases** | | **Expected Cases** | | **Time Period** | |
| --- | --- | --- | --- | --- | --- | --- | --- | --- | --- | --- | --- | --- |
| **Most Likely** | |  | |  | |  | |  | |  | |  |
| 1 | 21,017.38 | | <0.001 | | 1.66 | | 239,884 | | 160,512.13 | | 2005/1/1 to 2009/4/30 | |
| **Secondary** | |  | |  | |  | |  | |  | |  |
| 2 | 7,296.70 | | <0.001 | | 2.63 | | 21,312 | | 8,222.76 | | 2011/7/1 to 2017/12/31 | |

LLR, Log Likelihood Ratio; RR, relative risk.

**Supplementary Table 3.** Top 5 active PTB clusters from FleXScan

|  | **LLR** | **p value** | **RR** | **Observed Cases** | **Expected Cases** | **Maximum Distance** |
| --- | --- | --- | --- | --- | --- | --- |
| **Most Likely** | |  |  |  |  |  |
| 1 | 973.47 | <0.001 | 2.71 | 2,604 | 959.66 | 495 km |
| **Secondary** | |  |  |  |  |  |
| 2 | 722.59 | <0.001 | 1.96 | 3,816 | 1,947.42 | 178 km |
| 3 | 579.7 | <0.001 | 1.35 | 12,492 | 9,283.22 | 255 km |
| 4 | 174.36 | <0.001 | 1.66 | 1,572 | 946.98 | 640 km |
| 5 | 119.76 | <0.001 | 1.21 | 65,88 | 5,454.16 | 183 km |

LLR, Log Likelihood Ratio; RR, relative risk.

**Table S4.** Smear positive PTB clusters from SaTScan

| **ID** | **LLR** | **P value** | **RR** | **Observed Cases** | **Expected Cases** | **Time Period** |
| --- | --- | --- | --- | --- | --- | --- |
| **Most Likely** | |  |  |  |  |  |
| 1 | 21,857.82 | <0.001 | 2.03 | 153,546 | 94511.43 | 2005/1/1 to 2011/4/30 |

LLR, Log Likelihood Ratio; RR, relative risk.

**Table S5.** Top 5 smear positive PTB clusters from FleXScan

| **ID** | **LLR** | **p value** | **RR** | **Observed Cases** | **Expected Cases** | **Maximum distance** |
| --- | --- | --- | --- | --- | --- | --- |
| **Most Likely** | |  |  |  |  |  |
| 1 | 302.92 | <0.001 | 1.84 | 1,884 | 1,022.51 | 233 km |
| **Secondary** | |  |  |  |  |  |
| 2 | 123.47 | <0.001 | 1.248 | 4,680 | 3,750.5 | 209 km |
| 3 | 66.57 | <0.001 | 1.241 | 2,820 | 2,272.97 | 282 km |
| 4 | 52.34 | <0.001 | 1.22 | 2,544 | 2,079.38 | 186 km |
| 5 | 21.83 | <0.001 | 1.25 | 912 | 729.006 | 448 km |

LLR, Log Likelihood Ratio; RR, relative risk.
